# Sampling approaches and geographic coverage in Mayi Kuwayu: the national study of Aboriginal and Torres Strait Islander wellbeing

**DOI:** 10.1186/s13104-024-06692-0

**Published:** 2024-01-17

**Authors:** Joanne Thandrayen, Jennie Walker, Janet Chapman, Raymond Lovett, Katherine A Thurber

**Affiliations:** National Centre for Epidemiology and Population Health, College of Health and Medicine, Australian National University, 54 Mills Road, Acton, ACT 2601 Australia

**Keywords:** Australian Aboriginal and Torres Strait Islander Peoples, Indigenous, Cohort, Surveys, Sampling methods, Geographic coverage

## Abstract

**Objective:**

The objective of this paper is to investigate the geographic distribution of participants in Mayi Kuwayu, the National Study of Aboriginal and Torres Strait Islander Wellbeing. The Mayi Kuwayu Study is the largest national longitudinal study of the health and wellbeing of Aboriginal and Torres Strait Islander adults (aged 16 years and over) in Australia. It is an Aboriginal-led and governed Study with embedded community engagement. The Study collects data through self-report questionnaires, using multiple sampling approaches: (1) a large-scale mail-out based on stratified random sampling; (2) convenience sampling; (3) snowball sampling; (4) voluntary sampling. A comparison of the geographic distribution of Mayi Kuwayu Study participants to that of the total Aboriginal and Torres Strait Islander population was also conducted.

**Results:**

A total of 9,843 people participated in the Mayi Kuwayu Study baseline survey from 2018 to 2022. Participants resided in all Australian States and Territories. The geographic distribution of participants broadly matched the total population distribution, with participants generally located on the east and south-east coast of Australia. Apparent differences in the geographic distribution were identified by sex and age group.

**Supplementary Information:**

The online version contains supplementary material available at 10.1186/s13104-024-06692-0.

## Introduction

Representative (probability-based) surveys are considered the gold standard for producing reliable population-level estimates [1]. However, for some populations, it is difficult for researchers to access a complete sampling frame from which to draw a probability-based sample. For example, the Medicare (Australia’s health insurance) Enrolment Database is considered the most complete sampling frame for Aboriginal and Torres Strait Islander peoples, but only includes approximately 65% of the total Aboriginal and Torres Strait Islander population [2].

Representative social and health surveys about the Aboriginal and Torres Strait Islander peoples are conducted by the Australian Bureau of Statistics (ABS) every 3–5 years at the household level. However, these data resources lack Indigenous data governance and have been critiqued as to value, trust, and participation by Aboriginal and Torres Strait Islander peoples [3]. A representative sample of Aboriginal participants at the sub-national level (South Australia) was obtained by [4] where their study design was considered as culturally appropriate but proved to be methodologically challenging and required high levels of commitment and resources. There has been a shift from probability-based surveys towards non-probability based survey methods such as convenience or snowball sampling in Aboriginal and Torres Strait Islander health surveys in Australia [5–9]. However, no studies to date have collected data on a national scale and also included Aboriginal and Torres Strait Islander peoples’ leadership, governance of data and community partnerships.

## Overview of the Mayi Kuwayu Study

The Mayi Kuwayu Study is community-controlled and was developed via extensive collaboration with Aboriginal and Torres Strait Islander peoples from around Australia [10]. The Study is overseen by an Aboriginal and Torres Strait Islander governance group to ensure Indigenous Data Sovereignty and that Aboriginal and Torres Strait Islander Data Governance principles, including self-determination and strengths-based research, are maintained in analysis of the data [11]. The governance group includes several peak Aboriginal and Torres Strait Islander health and research groups, including the National Aboriginal Community Controlled Health Organisation, and State and Territory affiliate organisations.

The Mayi Kuwayu Study was developed within a social epidemiology framework to enable investigation of associations between cultural practice and expression, social determinates of health, health behaviours, and health and wellbeing outcomes for Aboriginal and Torres Strait Islander peoples [12]. The Mayi Kuwayu Study team members consulted with a total of 197 participants in various communities across Australia between 2015 and 2017. Participants were from all states and territories (see [13] for details).

The Mayi Kuwayu Study collects data through self-report questionnaires, with the majority of baseline data collected in 2019 and follow-up surveys conducted approximately every three years, and planned data linkage with morbidity and mortality databases. All Aboriginal and Torres Strait Islander adults aged 16 years and over are eligible to participate. Participants can join the study at any time, and are not required to have completed the baseline survey to be eligible. To date, more than 11,000 Aboriginal and Torres Strait Islander people have participated in the Study.

In this paper, we investigate the geographic coverage of participants in the Mayi Kuwayu Study, overall and by key demographic characteristics. We also compare the geographic distribution of Mayi Kuwayu Study participants to that of the total Aboriginal and Torres Strait Islander population.

## Methods

### Sampling approaches in the Mayi Kuwayu Study

The Mayi Kuwayu Study recruited participants through multiple sampling approaches, with the aim of maximising the participant sample and enabling individual/community self-determination in participation. This has included: (1) a large-scale mail-out based on stratified random sampling; (2) convenience sampling; (3) snowball sampling; (4) voluntary sampling. Apart from the mail-out which was based on probability sampling, the remaining approaches were based on non-probability sampling. As such, the probability of being selected for participation in the Study was not random across the total Aboriginal and Torres Strait Islander population of Australia. Therefore, like many cohort studies, the Mayi Kuwayu Study sample is not representative of the entire Aboriginal and Torres Strait Islander population. The baseline sample includes an over-representation of women, older adults, and those residing in more urban areas, compared to the distribution in the total population [10].

#### Stratified random sampling

Initial planning was to mail surveys to a total of 200,000 Aboriginal and Torres Strait Islander adults (approximately 25% of the total Aboriginal and Torres Strait Islander population– of all ages– in June 2018) identified using stratified random sampling from Medicare Enrolment Database. The Mayi Kuwayu Study team applied to the Department of Human Services (DHS), now Services Australia, to seek permission to use the Medicare Enrolment Database. The database was stratified by age, sex, and remoteness. The sample aligned with the distribution of Aboriginal and Torres Strait Islander population distribution across age group (16–24; 25–34; 35–49; ≥50 years), sex (male; female), and remoteness (major cities; inner and outer regional areas; remote and very remote). DHS mailed surveys to individuals, randomly selected from the total pool of eligible persons in each age-sex-remoteness stratum. The survey pack included a prepaid return envelope, an eight-page questionnaire, and an information sheet. In addition to the paper survey, respondents were provided options to complete the survey online or via a free-call helpline. The survey packs were mailed on 30 October 2018. A preliminary postal mail-out of 20,000 surveys was used to test response rates across the age, sex, and remoteness strata. An overall response rate of 2.3% (456/20,000) was achieved to the preliminary mail-out, with the highest response rates observed in males aged ≥ 50 living in regional areas, males aged ≥ 50 living in major cities, females aged ≥ 50 living in regional areas, and females aged ≥ 50 living in major cities [2]. With the remaining 180,000 postal surveys, the Mayi Kuwayu Study team decided to completely sample the highest-responding strata in order to maximise total response, rather than pursuing the same stratified random sampling approach.

#### Convenience sampling

In addition to postal surveys being sent to people, a local Community Researcher was employed to recruit and assist participants who had limited English literacy or who wanted help filling out the survey, in Communities where there was a need [2].

#### Snowball sampling

Supplementary recruitment also involved recruitment of new participants via existing participants. A selection of participants who responded to the preliminary and second-stage mail-out were contacted to seek their support in passing on the survey to family and/or friend.

#### Voluntary sampling

Additional recruitment occurred through people volunteering to complete the survey. Study promotion (advertising via social media and through local community-controlled organisations and word of mouth) was undertaken. Any eligible person could complete the survey online or over the phone, or contact the Mayi Kuwayu Study team to request a paper survey.

Given that the above recruitment methods potentially enabled participants to complete the survey multiple times, baseline data was checked for duplicates based on name, address and other identifying information.

### Data sources

The data for the following analysis were from Mayi Kuwayu baseline survey (June 2018-December 2020). In that dataset (major release 3.0; final version at 1 June 2021), a total of 9,843 people participated in the study. Participants with missing information on demographics of interest (Table 1) were excluded from corresponding analysis as appropriate.

**Table 1:** Characteristics of participants in the Mayi Kuwayu baseline sample

Characteristics	n	%
**Sex**		
Male	3729	37.9
Female	5858	59.5
Other	11	0.1
Missing	245	2.5
**Age group**		
16–17	176	1.8
18–39	2787	28.3
40–59	3719	37.8
60 plus	2834	28.8
Missing	327	3.3
**State/Territory**		
New South Wales	3324	33.8
Victoria	949	9.6
Queensland	2644	26.9
South Australia	424	4.3
Western Australia	1092	11.1
Tasmania	503	5.1
Northern Territory	683	6.9
Australian Capital Territory	144	1.5
Missing	80	0.8
**Remoteness**		
Major Cities of Australia	4048	41.1
Inner Regional Australia	2817	28.6
Outer Regional Australia	1864	18.9
Remote Australia	405	4.1
Very Remote Australia	667	6.8
Missing	42	0.4
**Total**	9843	

National population estimates, overall and by geographic location, were obtained from the ABS predicted projections. These projections were based on the 2016 Census of Population and Housing and estimated the Aboriginal and Torres Strait Islander population from 2006 to 2031. The ABS publishes three main population projections (series A, B, and C) that represent high, medium, and low population growth scenarios. The ABS considered Series B as the most appropriate projection for many users and was employed in the current analysis. Projections from 2019 were used as the majority of Mayi Kuwayu participants responded in that year and the difference in population estimates across the three series for 2019 were minimal. The age group of 15–19 years and older were included in the ABS data, aligning as closely as possible to the Mayi Kuwayu inclusion criteria of 16 years and older.

### Geographic distribution of Mayi Kuwayu Study participants and the total population

To compare the geographic distribution of Mayi Kuwayu Study participants to that of the total Aboriginal and Torres Strait Islander population, we used the ABS Australian Statistical Geography Standard (ASGS) Indigenous Structure (2016). This structure consists of three geographic units: Indigenous Locations, Indigenous Areas, and Indigenous Regions [14]. The current paper compares the largest geographic unit, Indigenous Region. This is “loosely based on the former Aboriginal and Torres Strait Islander Commission boundaries“ [14] and includes 37 geographical units defined for statistical and analytical purposes.

The home address of Mayi Kuwayu Study participants was geocoded by an external provider (Callpoint Spatial) into the geographic coordinates’ longitude and latitude. These coordinates were mapped onto Indigenous Regions as defined by the ABS ASGS Indigenous Structure (2016).

The ABS provide predicted projection data of the Aboriginal and Torres Strait Islander population only at the Indigenous Region level. Therefore, this paper restricts the analysis to Indigenous Regions to ensure comparability with national data.

### Statistical analysis

We presented descriptive statistics on the demographic characteristics of those 9,843 participants by sex (male/female/other), age group (16–17; 18–39; 40–59; ≥60 years), state/territory (New South Wales; Victoria; Queensland; South Australia; Western Australia; Tasmania; Northern Territory; Australian Capital Territory), and remoteness (major cities; inner regional; outer regional; remote; very remote). Data visualisations were created to show: (1) the geographic distribution of the Mayi Kuwayu participants across Australia and (2) how this distribution varied by sex and age group.

The number of Mayi Kuwayu participants was summed by Indigenous Region and the total number of people by Indigenous Region was computed in the ABS national data. Percentages of Aboriginal and Torres Strait Islander peoples by Indigenous Region at a national level were compared to those of the Mayi Kuwayu Study by Indigenous Region using Z-tests for difference in proportions wherever sufficient data was available (where the conditions of np > 5 and n(1-p) > 5 were satisfied; n = sample size; p = proportion). A significance level of 5% was specified for statistical testing.

## Results

The majority of Mayi Kuwayu Study baseline participants were female (59.5%) and aged 40 years and over (66.6%) (Table 1). The majority of respondents lived in New South Wales (33.8%) and Queensland (26.9%); with 69.8% residing in major cities and inner regional areas.

**Fig. 1:**
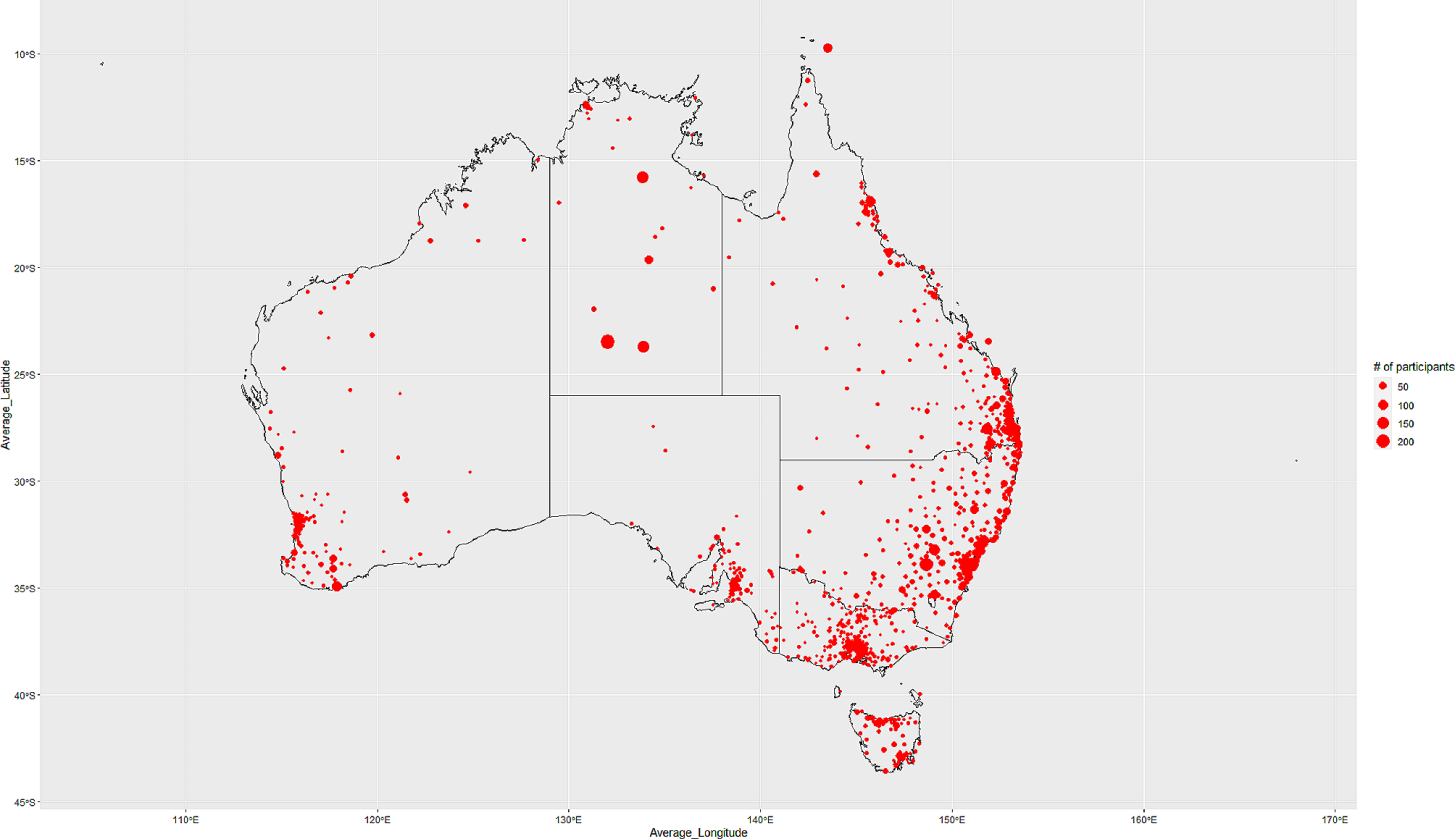
Geographic distribution of the Mayi Kuwayu cohort. Note: Figure 1 excluded 104 participants with missing postcode information (*n* = 9,739)

Figure 1 shows the geographic distribution of the Mayi Kuwayu cohort. All States/Territories were represented in Mayi Kuwayu baseline survey participation. In general, participants were mainly located on the east and south-east coast of Australia.

Figures S1 and S2 (Additional file 1) show the geographic distribution of the Mayi Kuwayu cohort by sex and age group respectively. The visualisations indicated that the distribution varied by sex, with more females located in the central part of Victoria and on the coast of Queensland. In New South Wales, the distribution seemed to be similar across both sex. Likewise, the visualisations indicated that the distribution also varied by age group, with older participants (aged ≥ 60) mostly located on the coast of New South Wales. Participants aged 40–59 seemed to be equally dispersed across New South Wales, Victoria, and Queensland. Younger participants (aged < 40) appeared to be located across all states.

A total of 99.6% (9,804/9,843) of Mayi Kuwayu Study participants had an Indigenous Region geocoded. The number of Mayi Kuwayu Study participants per Indigenous region ranged from < 5 to 1185 with a mean of 258.0 (SD = 302.6) and median of 143 (IQR = 307).

Table S1 (Additional file 1) shows a comparison between coverage of Indigenous Regions in the Mayi Kuwayu Study and coverage of Indigenous Regions according to national population estimates. Participants from Norfolk Island were excluded from this comparison as the ABS population projections did not include residents of Norfolk Island. Sufficient data was available for comparisons in approximately half of the Indigenous Regions investigated. For those Indigenous Regions, we generally found no statistically significant difference. We noted an over-representation of Mayi Kuwayu Study participants compared to the population distribution in one Indigenous Region (*p*-value < 0.05). The geographic distribution of Mayi Kuwayu Study participants thus approximately matched the geographic distribution of the total Aboriginal and Torres Strait Islander population (according to ABS population estimates).

## Discussion

The Mayi Kuwayu Study collected data on a national scale and included extensive collaboration with Aboriginal and Torres Strait Islander peoples and communities around Australia. As such it is a unique data resource which facilitates analyses at the national level. The Mayi Kuwayu Study has recruited participants through multiple sampling approaches, including stratified random sampling, convenience sampling, snowball sampling, and voluntary sampling. While the preliminary mail-out used stratified random sampling, the remaining approaches were based on non-probability sampling, and thus the probability of being selected for participation in the study was not random across the total Aboriginal and Torres Strait Islander population of Australia. Therefore, the Mayi Kuwayu Study sample is not intended to be representative of the entire Aboriginal and Torres Strait Islander population.

The current paper investigated the geographic coverage of participants in the Mayi Kuwayu Study, overall and by key demographic characteristics, resulting from the use of multiple sampling approaches. A comparison between the geographic distribution of the total sample of Mayi Kuwayu Study participants and that of the total Aboriginal and Torres Strait Islander population found that overall, the geographic coverage of participants in the Mayi Kuwayu Study was broadly similar to that of the total Aboriginal and Torres Strait Islander population in Australia. However, some potential differences were observed in the distribution of participants by sex and age group based on visual examination.

Overall, the current article provides valuable insights into the potential value of community-controlled, non-randomised studies such as the Mayi Kuwayu Study. The findings of the study have implications for researchers and policymakers seeking to understand the health and wellbeing of Aboriginal and Torres Strait Islander peoples and to develop policies and programs that are culturally appropriate and responsive to their needs.

### Electronic supplementary material

Below is the link to the electronic supplementary material.

**Additional file 1: Figure S1**. Geographic distribution of the Mayi Kuwayu cohort by sex; **Figure S2**. Geographic distribution of the Mayi Kuwayu cohort by age group; **Table S1**. Comparison between coverage of Indigenous Regions

## Data Availability

The dataset analysed during the current study is available on application to the Mayi Kuwayu Study Data Governance Committee. This governance body oversees and approves applications for data use in order to maintain Indigenous data sovereignty and the confidentiality of participants, and to ensure appropriate use of the Mayi Kuwayu Study data. The data application process is detailed here: mkstudy.com.au/ overview/.

## References

[1] Kruskal W, Mosteller F, Representative Sampling IV. The history of the Concept in statistics, 1895–1939. Int Stat Rev. 1980;48(169– 95).

[2] Wright A, Thurber KA, Yap M, Du W, Banks E, Walker J (2020). Who responds? An examination of response rates to a national postal survey of Aboriginal and Torres Strait Islander adults, 2018–2019. BMC Med Res Methodol.

[3] Eckford-Williamson B, Prehn J, Walter M, Lovett R, Bodkin-Andrews G, Maher B (2021). Indigenous peoples and the Australian census: value, trust, and participation. Australian Popul Stud.

[4] Marin T, Taylor AW, Grande ED, Avery J, Tucker G, Morey K (2015). Culturally appropriate methodology in obtaining a representative sample of South Australian Aboriginal adults for a cross-sectional population health study: challenges and resolutions. BMC Res Notes.

[5] Heckathorn DD, Respondent-driven Sampling II (2002). Deriving valid population estimates from chain-referral samples of hidden populations. Soc Probl.

[6] Cunningham J, O’Dea K, Dunbar T, Weeramanthri T, Zimmet P, Shaw J (2006). Study protocol-diabetes and related conditions in urban indigenous people in the Darwin, Australia region: aims, methods and participation in the DRUID Study. BMC Public Health.

[7] Hewitt B (2012). The longitudinal study of Indigenous children: implications of the study design for analysis and results.

[8] Ward J, Bryant J, Wand H, Kaldor J, Delaney-Thiele D, Worth H (2016). Methods of a national survey of young Aboriginal and Torres Strait Islander people regarding sexually transmissible infections and bloodborne viruses. Aust N Z J Public Health.

[9] Lee KSK, Fitts MS, Conigrave JH, Zheng C, Perry J, Wilson S (2020). Recruiting a representative sample of urban South Australian Aboriginal adults for a survey on alcohol consumption. BMC Med Res Methodol.

[10] Lovett R, Brinckley M-M, Phillips B, Chapman J, Thurber KA, Jones R (2020). The beginning it was our people’s law. What makes us well; to never be sick. Cohort profile of Mayi Kuwayu: the National Study of Aboriginal and Torres Strait Islander Wellbeing. Aust Aborig Stud.

[11] Maiam nayri Wingara Indigenous Data Collective and Australian Indigenous Governance Institute. Indigenous Data Sovereignty– Data for Governance: Governance of Data: Briefing Paper 2018. https://www.maiamnayriwingara.org/research Accessed on 16 March 2023.

[12] Jones R, Thurber KA, Chapman J, D’Este C, Dunbar T, Wenitong M (2018). Study protocol: our cultures count, the Mayi Kuwayu Study, a national longitudinal study of Aboriginal and Torres Strait Islander wellbeing. BMJ Open.

[13] Bourke SC, Chapman J, Jones R, Brinckley M-M, Thurber KA, Calabria B (2022). Developing Aboriginal and Torres Strait Islander cultural indicators: an overview from Mayi Kuwayu, the National Study of Aboriginal and Torres Strait Islander Wellbeing. Int J Equity Health.

[14] Australian Bureau of Statistics. 1270.0.55.002 - Australian Statistical Geography Standard (ASGS): Volume 2 - Indigenous Structure, July 2016. https://www.absgov.au/ausstats/abs@nsf/mf/1270055002 Accessed 19 Feb 2023.

